# Characterization and application of a lactate and branched chain amino acid metabolism related gene signature in a prognosis risk model for multiple myeloma

**DOI:** 10.1186/s12935-023-03007-4

**Published:** 2023-08-14

**Authors:** Zhengyu Yu, Bingquan Qiu, Hui Zhou, Linfeng Li, Ting Niu

**Affiliations:** 1 Department of Hematology, West China Hospital, Sichuan University, Chengdu, 610041 Sichuan China; 2 Department of Biochemistry and Biophysics, School of Basic Medical Sciences, Peking University Health Science Center, Beijing, China

**Keywords:** Multiple myeloma, Prognosis, Lactate, Branched-chain amino acids, Tumor microenvironment, Drug prediction

## Abstract

**Background:**

About 10% of hematologic malignancies are multiple myeloma (MM), an untreatable cancer. Although lactate and branched-chain amino acids (BCAA) are involved in supporting various tumor growth, it is unknown whether they have any bearing on MM prognosis.

**Methods:**

MM-related datasets (GSE4581, GSE136337, and TCGA-MM) were acquired from the Gene Expression Omnibus (GEO) database and the Cancer Genome Atlas (TCGA) database. Lactate and BCAA metabolism-related subtypes were acquired separately via the R package “ConsensusClusterPlus” in the GSE4281 dataset. The R package “limma” and Venn diagram were both employed to identify lactate-BCAA metabolism-related genes. Subsequently, a lactate-BCAA metabolism-related prognostic risk model for MM patients was constructed by univariate Cox, Least Absolute Shrinkage and Selection Operator (LASSO), and multivariate Cox regression analyses. The gene set enrichment analysis (GSEA) and R package “clusterProfiler"were applied to explore the biological variations between two groups. Moreover, single-sample gene set enrichment analysis (ssGSEA), Microenvironment Cell Populations-counter (MCPcounte), and xCell techniques were applied to assess tumor microenvironment (TME) scores in MM. Finally, the drug’s IC50 for treating MM was calculated using the “oncoPredict” package, and further drug identification was performed by molecular docking.

**Results:**

Cluster 1 demonstrated a worse prognosis than cluster 2 in both lactate metabolism-related subtypes and BCAA metabolism-related subtypes. 244 genes were determined to be involved in lactate-BCAA metabolism in MM. The prognostic risk model was constructed by CKS2 and LYZ selected from this group of genes for MM, then the prognostic risk model was also stable in external datasets. For the high-risk group, a total of 13 entries were enriched. 16 entries were enriched to the low-risk group. Immune scores, stromal scores, immune infiltrating cells (except Type 17 T helper cells in ssGSEA algorithm), and 168 drugs’IC50 were statistically different between two groups. Alkylating potentially serves as a new agent for MM treatment.

**Conclusions:**

CKS2 and LYZ were identified as lactate-BCAA metabolism-related genes in MM, then a novel prognostic risk model was built by using them. In summary, this research may uncover novel characteristic genes signature for the treatment and prognostic of MM.

**Supplementary Information:**

The online version contains supplementary material available at 10.1186/s12935-023-03007-4.

## Background

Multiple myeloma (MM), a plasma cell malignancy characterized by abnormal expansion of clonal plasma cells in the bone marrow, is the second most common hematological tumor in adults [1]. Over the past two decades, the emergence of new drugs and treatments have greatly improved the response rate and survival rate of patients with multiple myeloma [2, 3]. Nevertheless, high-risk MM patients still have disease recurrence and aggravation [4]. Due to the obvious heterogeneity in pathogenesis, clinical manifestations and prognosis of MM patients, it is of great research value and space to find more effective and reliable molecular markers for individualized treatment.

Lactate is the final metabolic waste product of glycolysis. Lactate production and accumulation in tumors can promote tumor growth and metastasis, and tumor cells can also take up and utilize lactate. High serum lactate level is associated with poor prognosis, overall survival, disease-free survival, and metastasis-free survival in breast cancer [5] and other cancers [6, 7]. Excessive lactate inhibits the normal function of T cells, resulting in poor anti-tumor effect of immunotherapy [8]. With the development of tumor metabolism and gene therapy research, lactate metabolism-related genes (LMRGs) have been considered as very valuable tumor therapeutic targets [9]. Lactate dehydrogenase (LDH) is the enzyme responsible for the reciprocal conversion of lactate and pyruvate. High serum LDH is associated with advanced disease characteristics and a poor survival rate of MM [10]. However, the relationship between lactate and MM remains unclear.

Branched chain amino acids (BCAAs), including leucine, isoleucine and valine, are essential amino acids of the body. The main metabolic pathway of BCAA is degradation metabolism. BCAAs is not only an important nutrient in the human body, which can provide essential raw materials for protein synthesis, but also participates in many physiological and pathological processes in the body through various metabolic pathways. At present, the biological mechanism between circulating branched-chain amino acids and tumor development is not clear. BCAAs promote the growth of cancer by participating in biosynthesis pathways and providing energy [11]. In addition, it can also help tumors escape the surveillance of immune cells [12]. The previous finding has shown that branched-chain amino acid transaminase 1 promotes the occurrence of mitochondria by activating the mTOR pathway, and then promotes the growth enhancement of breast tumor cells [13]. This was demonstrated in a number of studies that branched chain amino acid transaminase 1 may play a vital role in the prognosis of many tumors [12, 14, 15] and is considered as a prognostic biomarker of breast cancer [14]. However, the relationship between it and MM has not been studied so far.

Here, our research has demonstrated that the combined expression of lactate and branched chain amino acid metabolism genes is a reliable indicator of myeloma prognosis and can be utilized to guide treatment decisions. 256 samples were divided into high and low-risk groups by consensus clustering and the Kaplan-Meier (K-M) survival curves were drawn, which revealed that the survival of MM correlated with lactate metabolism and the related branched chain amino acids. In addition, the cytotoxic lymphocytes were significantly decreased high-risk groups, which could potentially reduce the anti-tumor effect. The risk score and independent prognosis of each clinical factor were investigated by univariate and multivariate regression. Importantly, the multiple metabolic genes (CKS2 and LYZ) correlated with markedly patient survival in MM were identified from this study. Temozolomide, alkylating agents, could be an alternative solution to treating myeloma patients whose branched chain amino acid and lactate levels are abnormal. In this study, a novel prognostic prediction model for multiple myeloma was developed, which was based on a gene signature related to lactate and chain amino acid metabolism.

## Materials and methods

### Collection of the date of the MM

GSE4581 (high purity bone marrow plasma cells from MM patients) and GSE136337 datasets (biopsy tissue of whole bone marrow) were sourced from the GEO database (https://www.ncbi.nlm.nih.gov/geo/). The prognostic signature was trained using the GSE4581 dataset, which contained data on 256 MM patients who had received TT2 prior to diagnosis. The GSE136337 dataset that contained 426 MM samples was utilized as a validation set for the assessment of the prognostic risk model. 787 samples (bone marrow tissue) and the clinicopathological information of MM were sourced from the TCGA databases (https://tcga-data.nci.nih.gov/) and also used to validate the prognostic signature. Each sample in these three datasets was with survival data.

Meanwhile, 13 lactate metabolism-relate genes (LMRG) and 27 BCAA metabolism-relate genes were acquired based on the GeneCards (https://www.genecards.org/) and Molecular Signatures Database (MSigDB, https://www.gsea-msigdb.org/gsea/msigdb/), respectively.

### Consensus clustering

Based on the expression of 13 LMRG, the R package “ConsensusClusterPlus” [16] was utilized to identify different lactate metabolism-related subtypes among the MM samples. Likewise, BCAA metabolism-related subtypes of MM samples were generated according to the expression patterns of 27 BCAA metabolism-related genes as well. Principal component analysis (PCA) was applied to validate these clustering results. Additionally, the R package “Survival” was utilized to compare the overall survival (OS) among different subtypes. Besides, the expression heatmap of related genes in different subtypes of MM was pictured.

### Acquirement of differentially expressed genes (DEGs) in different subtypes

The limma package [17] based on |log_2_FC|>0.5 and p.adjusted < 0.05 was used to screen DEGs among lactate metabolism-related subtype and BCCA metabolism-related subtype, respectively. The “ggplot2” was adopted to plot the volcanic maps and heatmaps [18] and “pheatmap” [19] to visualize DEGs. Moreover, Gene Ontology (GO) and Kyoto Encyclopedia of Genes and Genomes (KEGG) pathway enrichment analysis of DEGs was done with the “clusterProfiler” R package [16]. The “ggplot2” was utilized to display the outcome.

### Construction and validation of the prognostic risk model of MM

By using univariate Cox analysis of intersecting genes (lactate-BCAA metabolism-related gene) in the training set, the prognosis-related genes were acquired (*P <* 0.005) Subsequently, the most predictive prognostic genes were identified by LASSO [20] analysis and multivariate Cox analysis then sequentially. Based on the median risk score, the training set of MM patients was separated into two groups. Kaplan-Meier (KM) curves were then utilized to show the difference in OS between the two groups. The “survROC” was applied to display the ROC curves [21] to perform an assessment of the prognostic capability of prognostic risk model. At last, we validated the prognostic risk model in the external validation GSE136337 dataset and TGCA-MM.

### The relationship between the clinical characteristics and the risk scores

The following analyses were performed to explore the relationship between clinical characteristics and risk scores. The chi-square test was applied to identify clinical characteristics that were substantially different between the two groups. For clinical characteristics classified differentially in the two groups, survival analysis was carried out using K-M curves.

### Analysis of independent prognostic

To determine if clinicopathological characteristics and risk scores were independent predictive factors for MM patients, univariate and multifactorial Cox analyses were performed. The “rms” (Harrell Jr FE (2022). _rms: Regression Modeling Strategies_. R package version 6.3-0, <https://CRAN.R-project.org/package=rms>) was adopted to construct the nomogram to predict survival probability based on independent prognostic criteria.

### Biological differences between two groups

Based the following criteria: |log2FC|>0.5, P.Value < 0.05, the DEGs were acquired using the “limma”, and GO and KEGG analysis were adopted to the DEGs. Additionally, the enrichment pathways were investigated using GSEA.

### Tumor microenvironment analysis

The ssGSEA [22] was adopted to calculate The abundance of distinct immune cell infiltrations in all of the MM samples. Microenvironment Cell Populations-counter (MCPcounter), and xCell algorithms in order to appropriately analyze the tumor microenvironment of MM. Seven immunomodulators’ differential expression in the training set was assessed using the Wilcoxon rank-sum test.

### Drug prediction analysis

Using the “oncoPredict” R program, the therapeutic medicines for MM were predicted based on GDSC (https://www.cancerrxgene.org/) [23]. To compare the two groups’ differences in drug sensitivity, we adopted the Wilcoxon rank-sum test. Then, the characteristic gene’s protein structures were sourced from the PDB database (http://www.rcsb.org/), and AutoDock Tools was applied to calculate the protein hydrogenation and charge [24]. PubChemdatabase(https://www.ncbi.nlm.nih.gov/pccompound/) was applied for downloading the chemical structures of medicines’ active ingredients. The AutoDock tool was used to check the charge balance and rotatable bonds of tiny molecules. To produce docking energy, AutoDock Vina [25] ran docking simulations. To view the docked complexes, PyMol software [26] was lastly employed.

### Extraction of total RNA from spinal fluid samples and qRT-PCR

To confirm the expression pattern of the characteristic genes in MM more precisely, bone marrow fluids of 14 MM patients and 14 normal samples were used for this study. 500ul of each sample was taken separately, and all intracellular RNA was extracted by Trizol reagent, and the quality of the extracted RNA were detected by nanodrop (Thermo scientific). The extracted RNA was reverse transcribed to cDNA according to manufacture instructions to detect the following targets expression. The BlazeTaq™ SYBR® Green qPCR Mix2.0 kit (Genecopoeia) and the following reaction system were uutilized to perform qRT-PCR reactions next. Primer sequences are shown in Table S1. The CT values of each gene were counted, and the relative expression of characteristic genes was analyzed according to the 2-ΔCt method using GAPDH as the internal reference gene.

### Gene silencing

siRNAs and negative control were transfected into MM1.S cells by using INTERFERin® (Poly-Plus Corporation) reagent. After 48 h, the cells were harvested and used for rest experiments. The siRNA sequences are provided in Table S3.

### Cell proliferation analysis

Cells(2.0 × 10^4^) were transfected and seeded into 96-well plates. After 48 h of culturing, WST-8 solution (Enhanced Cell Counting Kit-8, 1:10) was added and incubated for two hours to measure cell proliferation. The OD value was ascertained by a microplate reader.

### Transwell Assay

For migration and invasion assays, cell suspensions of 3 × 10^5^ and 5 × 10^5^ cells in serum free medium were seeded on Transwell membranes (8 μm pore size, Costar, Corning Incorporated, NY, USA). The membranes were coated with or without Matrigel (BD Biosciences, NJ, USA) for the invasion assay or the migration assay. Following incubation, cells on the upper membranes were fixed and stained with crystal violet for 30 min. 10% FBS was added to the medium in the lower chambers. Subsequently, the migrated or invade cells were imaged using a microscope (Nikon, Tokyo, Japan). To quantify the cells that had migrated into the lower chambers within 24 h, FACSCalibur flow cytometer (BD Biosciences) was utilized.

### Apoptosis analysis

Cells were transfected with siRNA for 48 h, after which they were collected and centrifuged. Subsequently, the medium was removed and the cells were stained with annexin V/FITC and 7AAD (4 A Biotech). Finally, the cells were stored in the dark at room temperature for 15 min. Flow cytometry analyses were conducted by utilizing a FACSCalibur flow cytometer (BD Biosciences) to identify cell apoptosis. FlowJo software was used to analyze the data.

### Statistical analysis

All analyses were conducted utilizing R version 3.4.1 and its associated packages. Student’s t-test was utilized in this study to determine any discrepancies. The selection of statistical methods is outlined in the study methods. Statistical significance was determined at a maximum P value of 0.05 (*p < 0.05, **p < 0.01, ***p < 0.001, **** p < 0.0001). This study’s experimental data was repeated more than three times. GraphPad Prism 9 was utilized to analyze the differences between groups.

## Results

### Identification of lactate metabolism subtypes and branched-chain amino acid metabolic subtypes

**Fig. 1:**
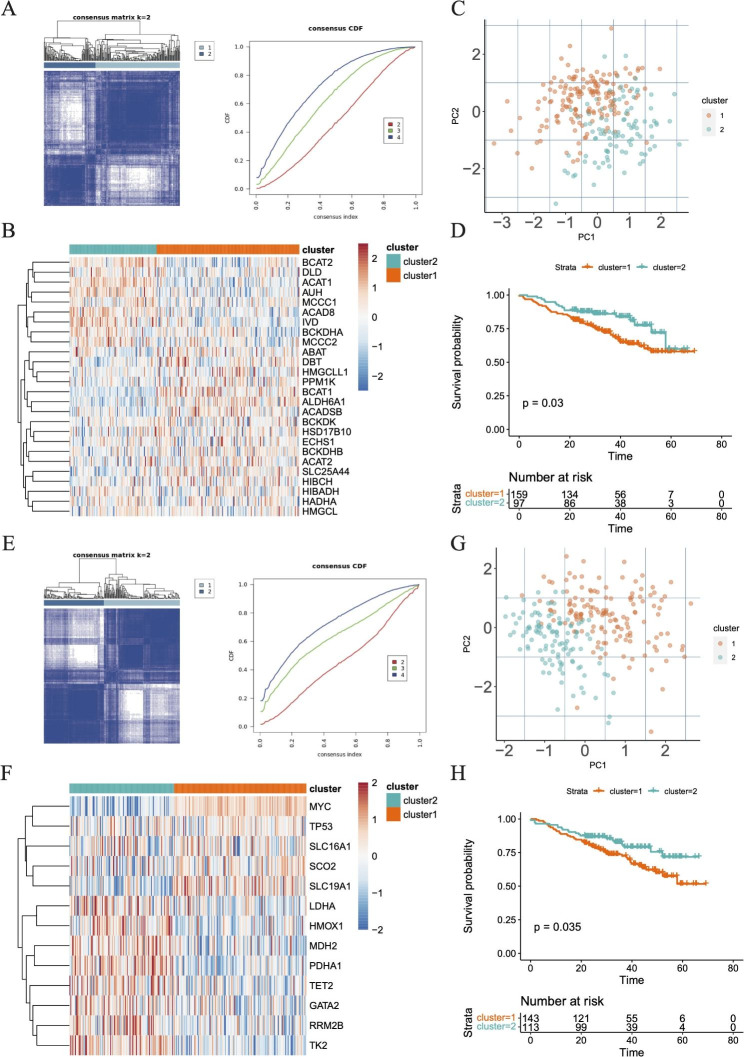
The identification of distinct subtypes of BCAA and LMRG metabolism. **A** Based on the cumulative distribution function (CDF) plot and consensus clustering matrix of consensus clustering with k valued 2 to 3, the intragroup correlations were the highest and the inter-group correlations were low when k = 2 (BCAA metabolism). **B** The expression of genes associated with BCAA metabolism in different subtypes. **C** Principal component analysis. **D** K-M survival analysis among the BCAA-related clusters. **E** Based on the CDF plot and consensus clustering matrix of consensus clustering with k valued 2 to 3, the intragroup correlations were the highest and the inter-group correlations were low when k = 2 (lactate metabolism). **F** The expression of genes associated with LMRG metabolism in different subtypes. **G** Principal component analysis. **H** K-M survival analysis among the LMRG-related groups

It could be seen that the best clustering results achieved when 256 MM patients were clustered into two molecular subtypes (k = 2) no matter based on the expression patterns of 27 BCAA metabolism-related genes (cluster 1 = 159, cluster 2 = 97) or 13 LMRG (cluster 1 = 143, cluster 2 = 113) (Fig. 1A and E). The expression of BCAA metabolism-related genes and LMRG among different subtypes were presented in Fig. 1B and F, correspondingly. The PCA results showed the reasonableness of these clustering results, exhibiting a good internal consistency and stability (Fig. 1C and G). In both BCCA metabolism-related subtypes and lactate metabolism-related subtypes, it was cluster 1 that had a poorer prognostic outcome than cluster 2 (Fig. 1D and H).

### Analysis of DEGs in different subtypes

**Fig. 2:**
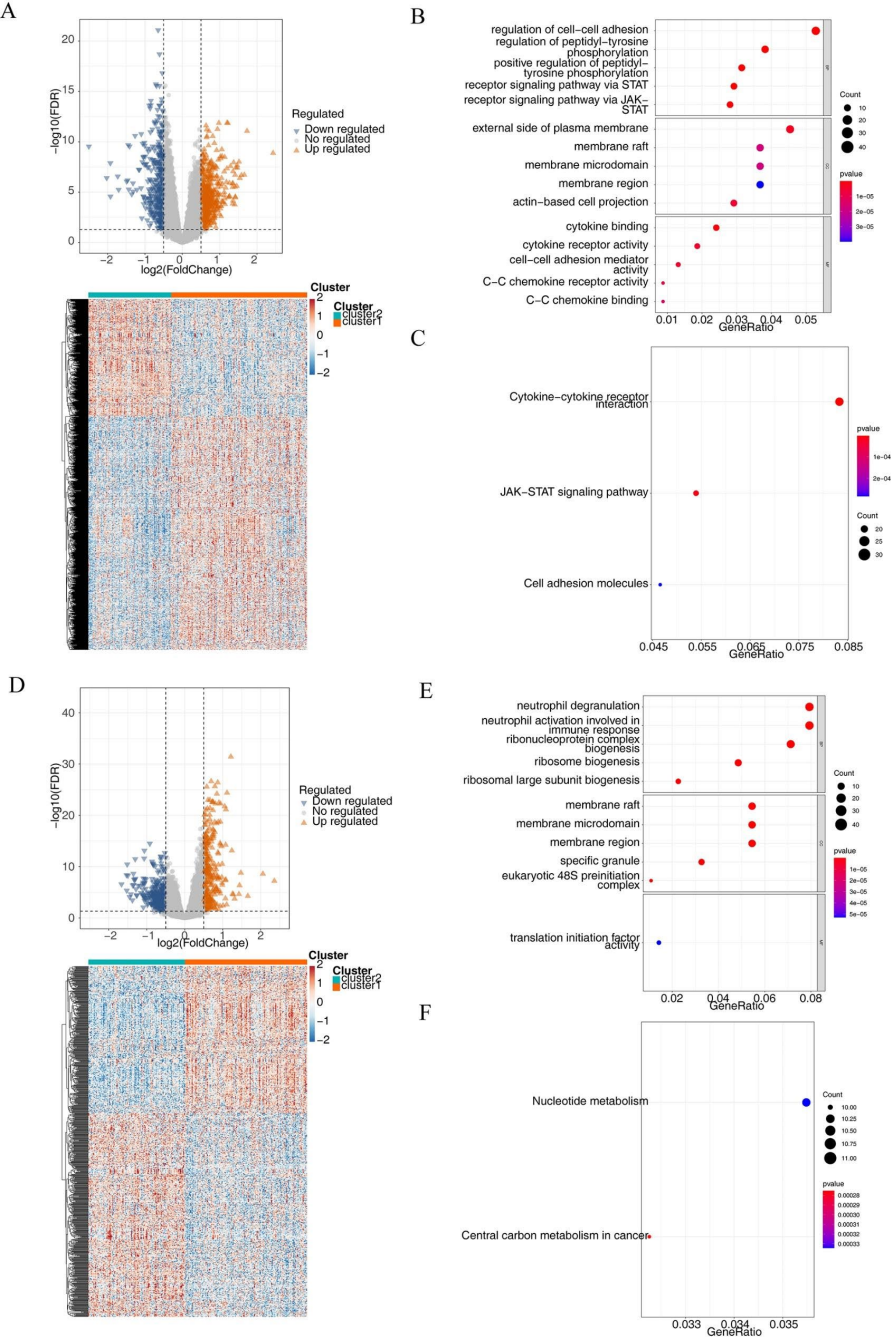
Differential expression analysis of BCAA and LMRG related subtypes. **A** Volcano plot and heatmap of DEGs among the BCAA groups. **B** GO and **C** KEGG terms enriched in BCAA DEGs. **D** Volcano plot and heatmap of DEGs among the LMRG groups. **E** GO and **F** KEGG terms enriched in LMRG DEGs.

Among the BCCA metabolism-related subtypes, 1079 DEGs were screened (718 genes up-regulated and 361 genes down-regulated) (Fig. 2A). These genes were connected to the control of peptidyl-tyrosine phosphorylation and the control of cell-cell adhesion in MM, according to the BP analysis. In the case of CCs, these DEGs were engaged in actin-based cell projection, membrane raft, and the external side of the plasma membrane. Regarding MF, These DEGs were involved in specific important functions, for example, C-C chemokine receptor activity and cytokine binding (Fig. 2B). In a further, KEGG pathway enrichment results revealed these DEGs were only linked to JAK-STAT signaling pathway, the Cytokine-cytokine receptor interaction, as well as Cell adhesion molecules (Fig. 2C). Among the lactate metabolism-related subtypes, 699 DEGs (294 genes with increased expression and 405 with decreased expression) were screened (Fig. 2D). The BP analysis revealed that these DEGs were linked to immune response-related neutrophil degranulation and activation. In the case of CCs, these DEGs were also engaged in the activity of membrane rafts. Regarding MF, these DEGs only involved in translation initiation factor activity (Fig. 2E). In particular, these genes were connected to central carbon metabolism and translation initiation factor activity in cancer, according to the KEGG pathway enrichment study (Fig. 2F).

### Great functionality of risk signature

**Fig. 3:**
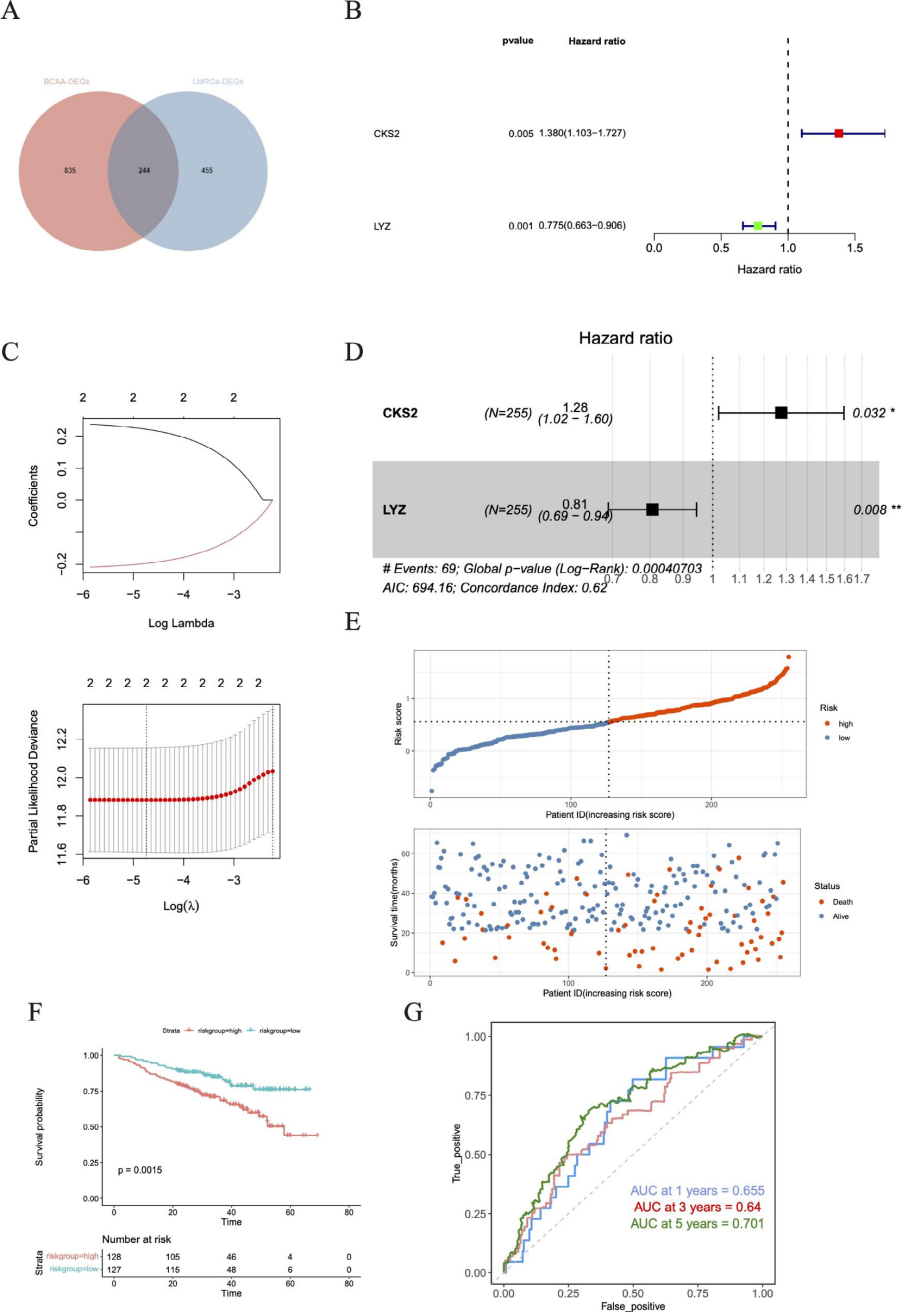
Analysis and assess the prognostic gene signature related to BCAA and LMRG associated with MM. **A** Venn diagram of BCAA and LMRG DEGs. **B** Forest plot of hazard ratios for 2 prognostic BCAA and LMRG related genes. **C** Cross-validation for tuning parameter selection in the LASSO model. **D** Forest plot of univariate Cox regression of OS related BCAA and LMRG related genes. **E** The distributions of risk score, survival status and expression profile of signature genes between the risk groups. **F** K-M survival analysis between the high- and low-risk groups. **G** ROC curve at 1-, 3- and 5-years of prognostic value of the prognostic index

A number of 244 intersecting genes were obtained by the Venn diagram (Fig. 3A), in which, only two genes (CKS2 and LYZ) were associated with MM prognosis with *P* < 0.05 and were selected as prognostic genes by univariate Cox regression analysis. (Fig. 3B). Lasso regression is a statistical method that avoids multicollinearity and overfitting in multiple regression models to obtain a more refined model. When lamda.min = 0.00874, the regression coefficients of these two prognostic genes were not 0 (Fig. 3C). To further screen for genes with the greatest prognostic value, multifactorial Cox regression was performed to investigate their effects, and CKS2 and LYZ were still selected as the characteristic genes to construct a prognostic risk model for MM patients (Fig. 3D). Risk score = 0.2214×CKS2–0.1970×LYZ. Based on median risk = 0.5591 (Fig. 3E), the MM patients were divided into the high and low-risk groups. The result of K-M curve revealed that the prognosis was better for lower risks. (Fig. 3F). The ROC curve showed that this risk score signature has an area under the ROC curve (AUC) of 0.655, 0.640,and 0.701 at 1, 3, and 5 years, respectively (Fig. 3G), indicating that this prognostic risk model has moderate performance (Fig. 3G). The prognostic risk model still had strong predictive power in TCGA-MM and GSE136337 datasets (Figure S1-2).

### Assessment of the prognostic risk model

**Fig. 4:**
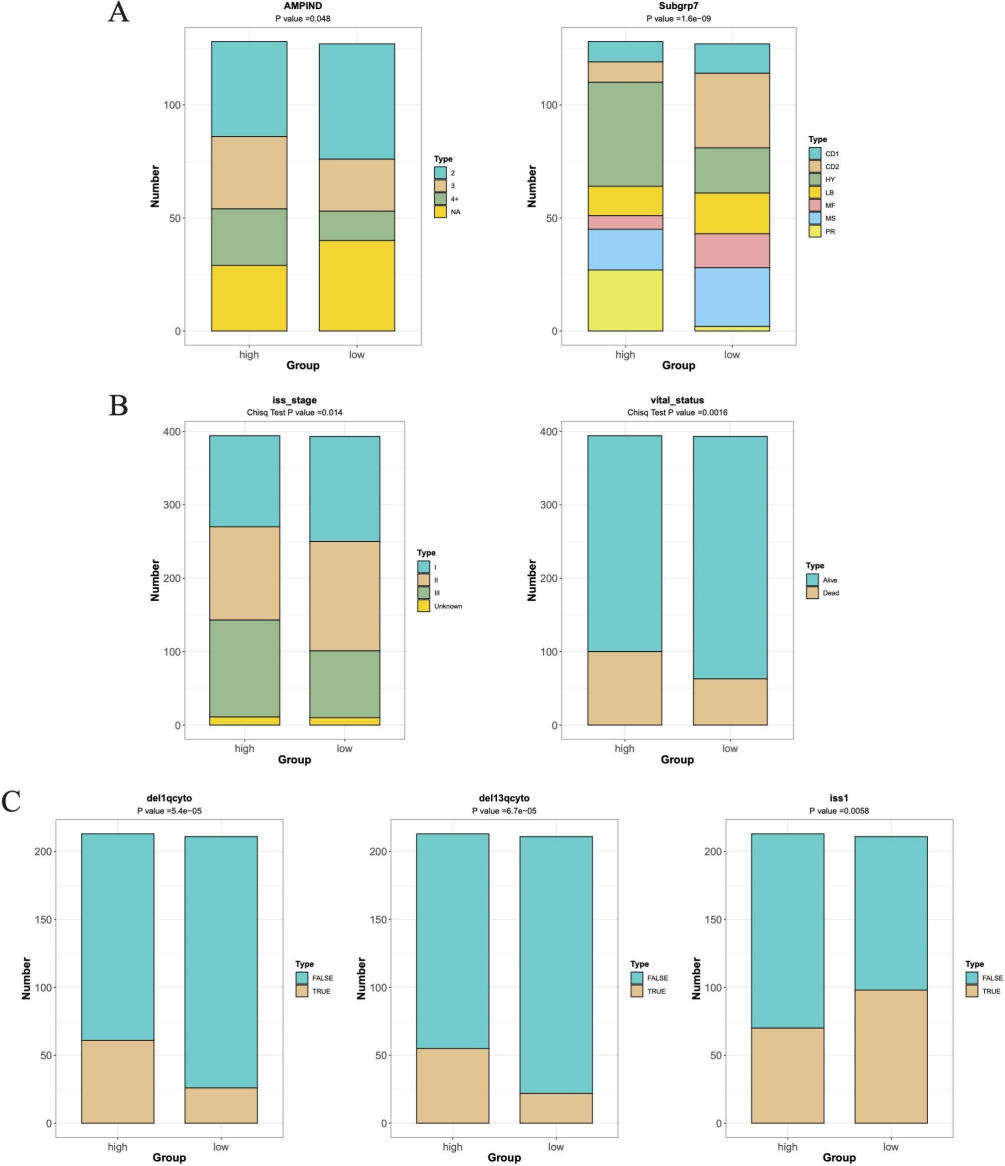
Assessment of the prognostic risk signature. **(A)** Subgrp7, AMPIND, and OS were evaluated in two risk groups. **(B)** The OS and iss in the TCGA dataset were evaluated in two risk groups. **(C)** The del1qcyto, del13qcyto, and iss in GSE136337 were evaluated in two risk groups

**Fig. 5:**
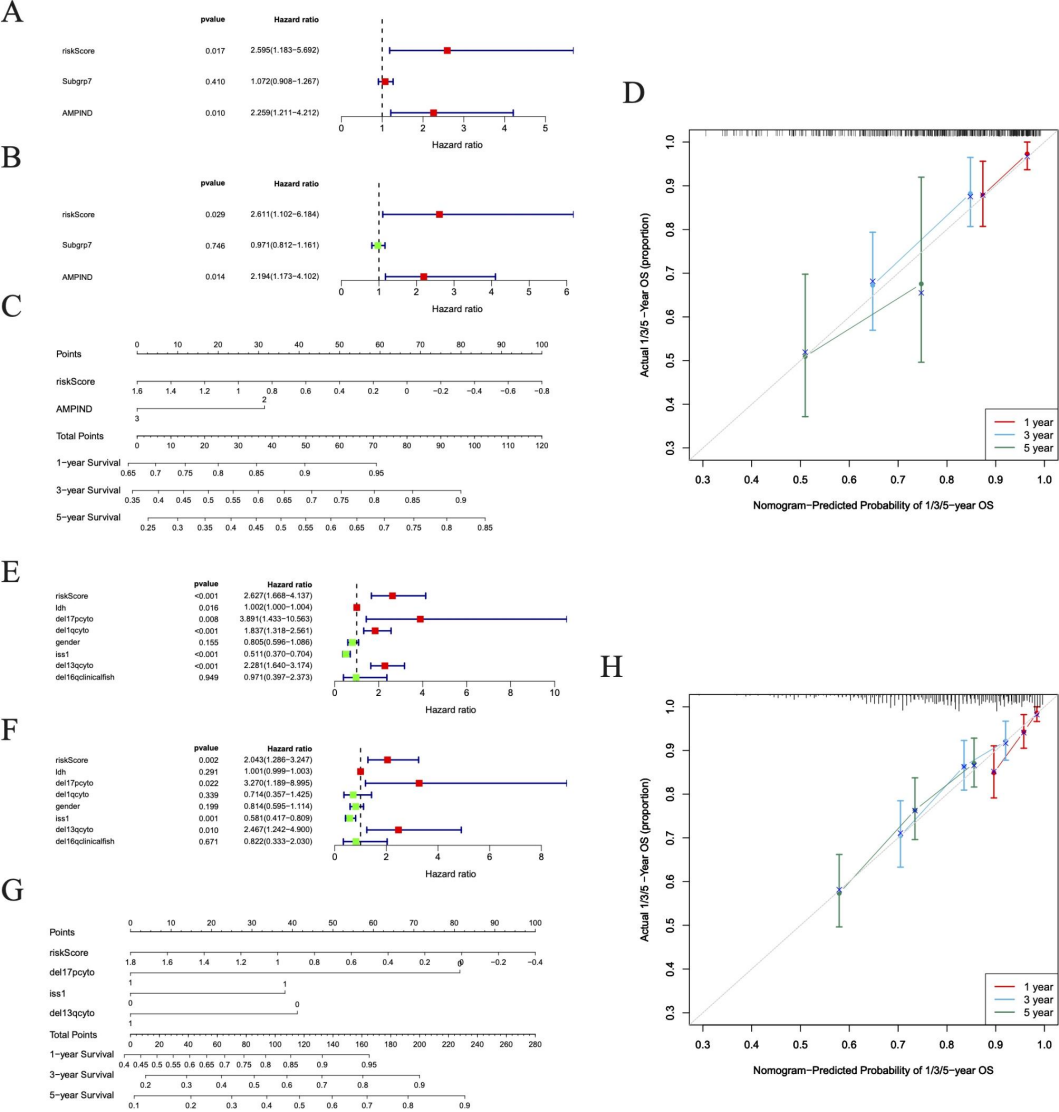
The Risk Score is an independent prognostic indicator. **A-B** Forest plot of hazard ratios for clinicopathological characteristics by Cox analysis in GSE4581 and GSE136337 dataset. **C-D** Nomogram was used to show the survival probability at 1-, 3- and 5-years in GSE4581 dataset. **E-F** Forest plot of hazard ratios for by Cox analysis in GSE4581 and GSE136337 dataset. **G-H** Nomogram was used to show the survival probability at 1-, 3- and 5-years in GSE136337 dataset

Subgrp7, AMPIND, and OS were significantly different (Fig. 4A). The OS and iss in the TCGA dataset were different (Fig. 4B). The del1qcyto, del13qcyto, and iss in GSE136337 were significantly different (Fig. 4C). In the training set and TCGA-MM dataset, there were survival differences in Subgrp7-CD1, and differences in OS, iss1, gender, del17pcyto, and del16qclinicalfish in GSE136337 (Figure S3). In the training set and GSE136337 dataset, risk score and AMPIND were independent predictive indicators (Fig. 5A and B). The nomogram were constructed based on independent prognostic factors to assess 1, 3, and 5-year OS in relation to risk score and AMPIND (Fig. 5C). (The slope of the calibration curve converges with 1, proving that the value of the nomogram was a good predictive tool for MM prognosis (Fig. 5D). Similarly, the risk score, del17pcyto, iss1, and del13qcyto in the validation set GSE136337 were independent prognostic factors for MM (Fig. 5E F), and the nomogram in the validation set still had good performance (Fig. 5G H). This showed the importance of the risk score for MM.

#### Biological differences between the two risk groups

**Fig. 6:**
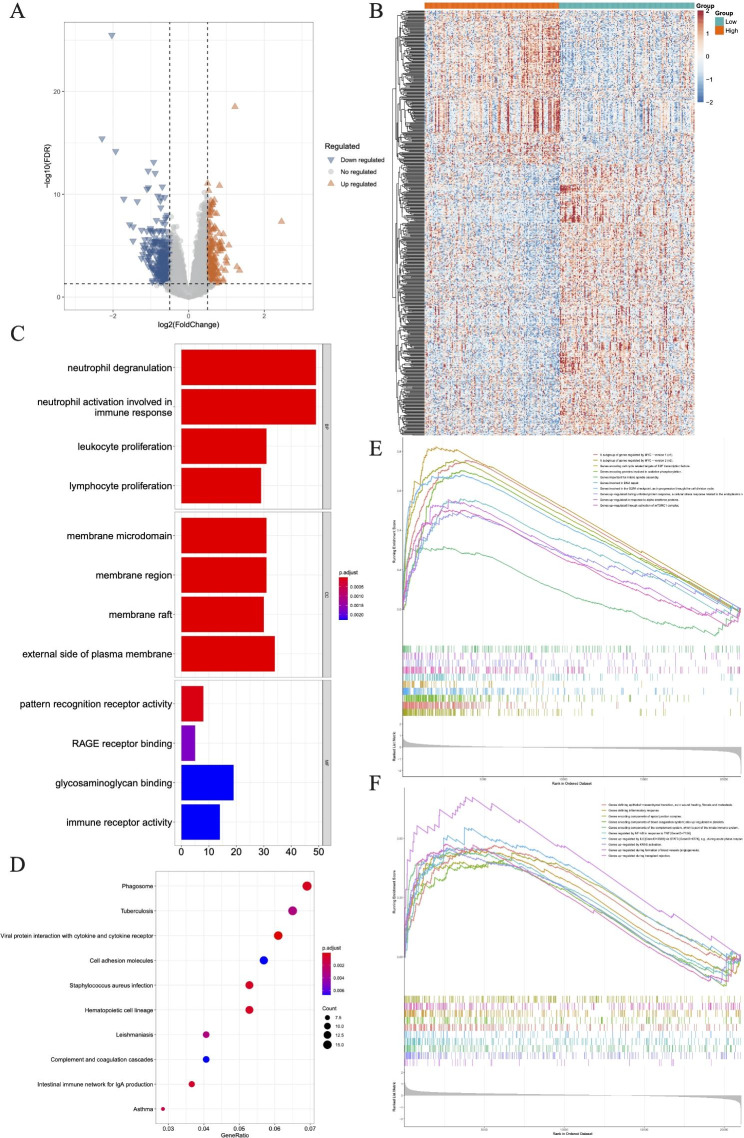
Differential expression analysis of high- and low-risk groups. **A-B** Volcano plot and heatmap of DEGs between the two groups. **C-D** The top 4 GO BP MF and KEGG terms of up- and downregulated DEGs in the two groups. **E** Result of GSEA analysis in the training set in the high-risk group. **F** Result of GSEA analysis in the training set in the low-risk group

Following two risk groups were generated, the differentially expressed analysis between groups were screened for DEGs as well, in which a total of 559 DEGs with |log_2_FC|>0.5, *P*.Value < 0.05 (202 DEGs upregulated and 357 DEGs downregulated) were obtained (Fig. 6A and B). Leukocyte proliferation and Neutrophil degranulation are the main biological processes enriched in these DEGs (Fig. 6C). Viral protein interaction with cytokine and cytokine receptor pathways might be related to MM (Fig. 6D). BGSEA results indicated that the DEGs in the high-risk group were primarily enriched in Genes encoding cell cycle-related targets of E2F transcription factors, a subgroup of genes that are regulated by MYC (Fig. 6E). While KRAS activation, angiogenesis, and genes encoding components of the complement system were the three main mechanisms by which the DEGs in the low-risk group were enriched (Fig. 6F).

### Correlation analysis of risk scores and immune microenvironment

**Fig. 7:**
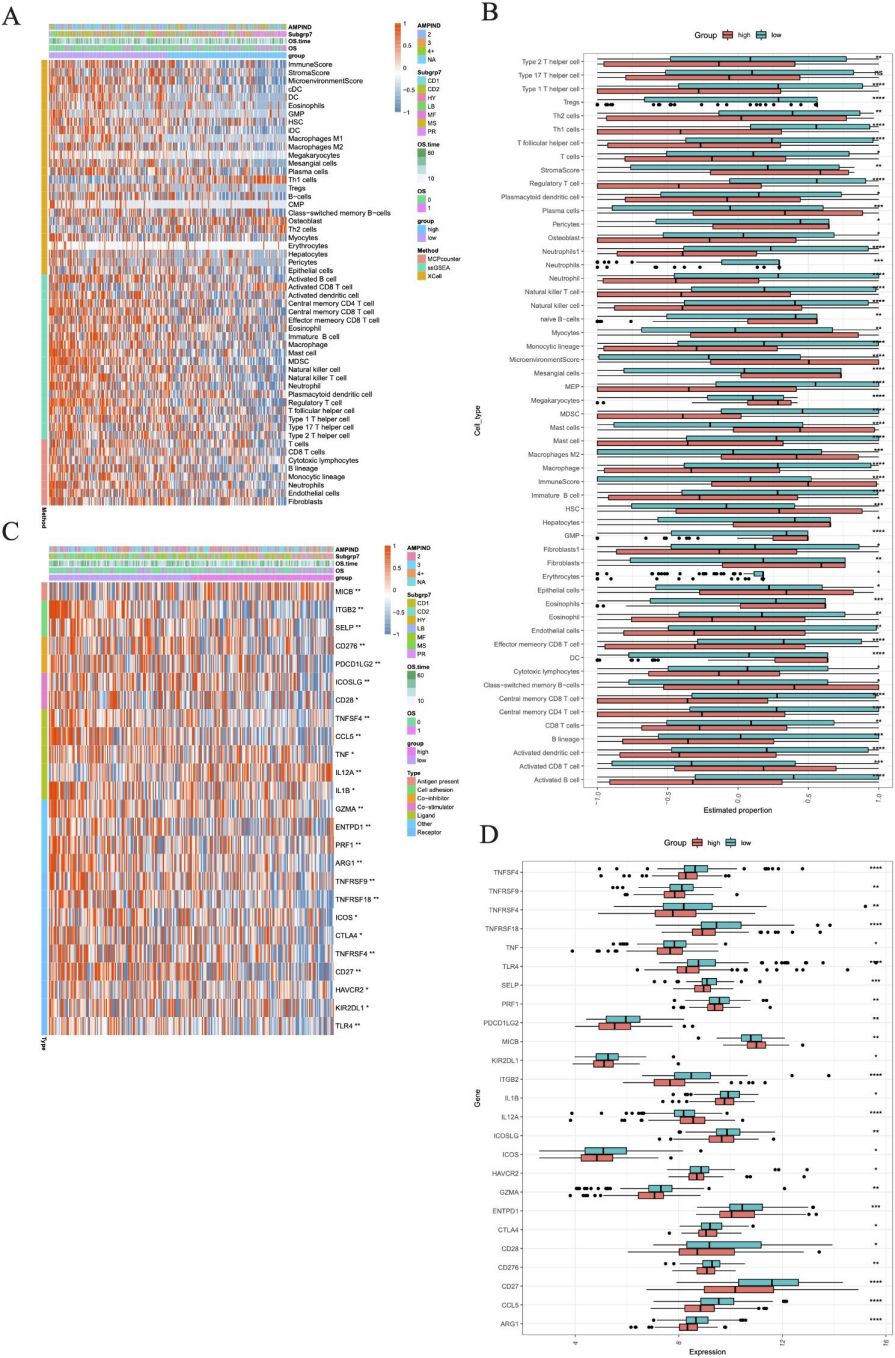
Analysis of immune infiltration and immunomodulators expression in high- and low-risk groups. **A** Heatmap of immune cell subset proportions. **B** Result of the infiltrating score of immune infiltrating cells, immune scores and stromal scores in two groups. **C-D** The immunomodulators genes expression in the two groups. (*, p < 0.05; **, p < 0.01; ****, p < 0.0001; vs. Low-risk group)

Analysis of the 3 algorithms revealed the abundance of various immune cell infiltrates in the MM samples in the training set, and the results showed that only Type 17 T helper cells in ssGSEA were not significant in the high- and low-risk groups, while other immune infiltrating cells, immune scores and stromal scores enhanced immune-related features with increasing scores (Fig. 7A and B). Not only that, immunomodulators were all significantly differentially expressed in the high and low risk groups (Fig. 7C and D). Among them, PDCD1LG2, KIR2DL1 and ICOS were expressed at lower levels in the high-risk group.

### The analysis of MM-related drug prediction

**Fig. 8:**
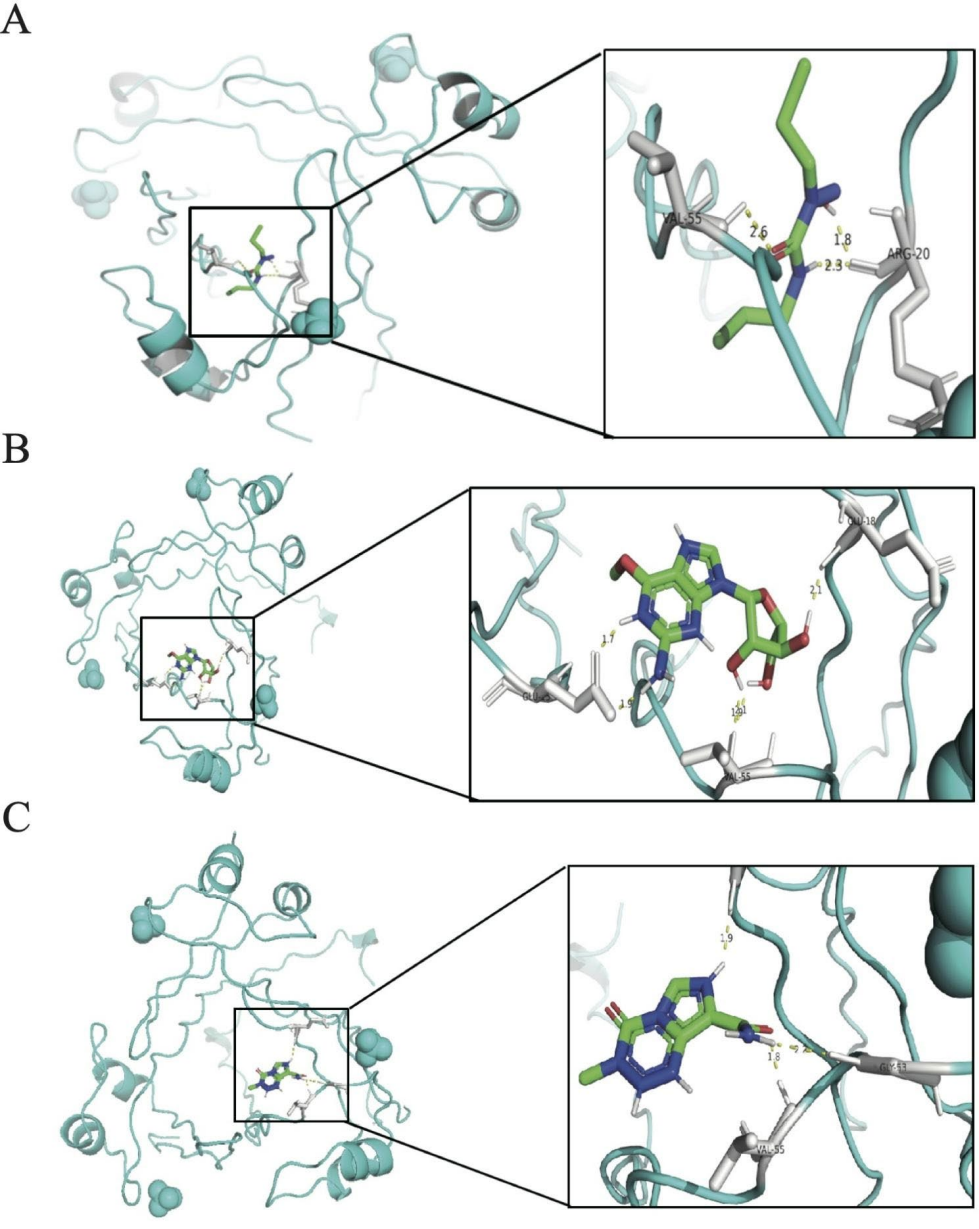
The analysis of MM-related drug prediction. **A-C** Top 3 drug interaction conformation and interaction of CKS2 The top 3 drug sensitivity drug and the molecular docking of the proteins encoding the CKS2 (Carmustine_1807 **(A)**, Nelarabine_1814 **(B)** and Temozolomide_1375 **(C)**), respectively

The “oncoPredict” R program was used to compute IC50 for each MM patient between the two groups using the GDSC database. This process produced 168 medicines with significantly different IC50s in the two groups (Table S2). The IC50 was lower in the high-risk group, indicating that high-risk patients are better suited for drug therapy. The top 3 drugs with the highest drug sensitivity (Carmustine_1807, Nelarabine_1814, and Temozolomide_1375) and the molecular docking of the proteins encoding the CKS2 and LYZ were explored using AutoDock (Fig. 8A,B,C). The docking fractions between the characteristic genes and the three drugs was less than − 1.2 k/mol, indicating that the three drugs could perfectly interact with the characteristic genes to influence the development of MM. For example, CKS2 interacted with Carmustine_1807, Nelarabine_1814, and Temozolomide_1375 through 3, 5, and 3 hydrogen bonds, respectively, and the docking fractions were − 5.71, -7.88 and − 5.73 kcal/mol, respectively. We verified that Nelarabine, Carmustine and Temozolomide could effectively enhance apoptosis in MM cells, and that Temozolomide could significantly inhibit the invasion and migration of MM cells (Figure S4).

### Expression and validation of CKS2 and LYZ

**Fig. 9:**
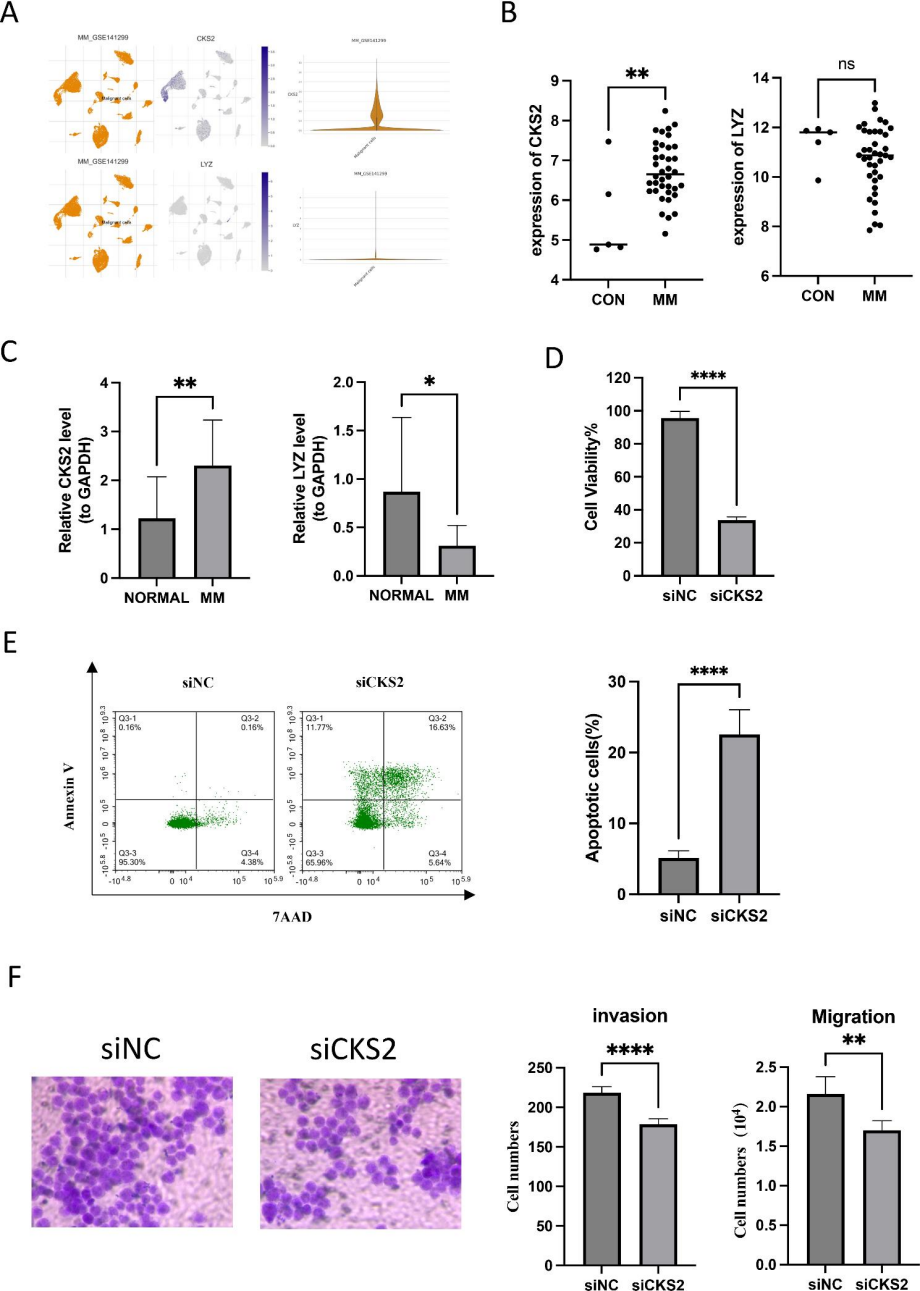
Validation of the expression of the 2-gene signature. **A** T-SNE describes the expression profiles of CKS2 and LYZ in the single cells obtained from myeloma tissues. Every point on the graph represents a single cell. The plot can be downloaded from the CancerSEA database. **B** BCKS2 and LYZ mRNA expression in newly diagnosed, non-treated multiple myeloma patients were measured by microarray from GSE39754. **C** The relative expression levels of the 2 genes compared with GAPDH in normal (n = 14) and MM (n = 14) samples (*, p < 0.05; **, p < 0.01). **D** Knockdown of CKS2 reduced the cell viability of MM1.S myeloma cell line. **E** Knockdown of CKS2 increased the cell apoptosis of MM1.S myeloma cell line. **F** Invasion and migration ratio of MM cell toward two groups through Transwell membranes (5-mm pore size) were assessed. Independent experiments were performed 3 times. n = 5 per group (**, p < 0.01; ****, p < 0.0001; vs. CONTROL; ns, no significance)

We made use of the Tumor Immune Single-cell Hub (TISCH) database and employed Uniform Manifold Approximation and Projection (UMAP) to examine the expression levels of CKS2 and LYZ in single cells obtained from myeloma tissues (Fig. 9A). Data from the Gene Expression Omnibus (GEO) dataset was obtained for both MM samples and normal tissue samples, with the aim of using CKS2 and LYZ in microarray analysis. Our results revealed that CKS2 expression was highly expressed in MM samples (Fig. 9B). After examining the expression of CKS2 and LYZ, we found that the CKS2’expression was much higher in MM than in normal bone marrow fluids, while LYZ was just the opposite (Fig. 9C). To verify the carcinogenic and invasive capacity of CKS2, we generated stably silenced CKS2 cell lines using MM1.S cells and assessed their viability. CKS2 silencing promotes cell apoptosis (Fig. 9E), and inhibits the proliferation, migration, and invasion of A172 cells (Fig. 9D, F). This result demonstrated that CKS2 could be used as characteristic genes for MM prognosis and treatment.

## Discussion

MM is a malignant tumor that is caused by the overgrowth of plasma cells. Elevated aerobic glycolysis levels during tumor metabolism produce a substantial amount of lactate, thus facilitating tumor growth [27, 28]. The metabolism of BCAAs can also affect multiple cancer characteristics and serve as an indicator of disease progression [14, 29]. Research has demonstrated that the assessment of LMRG can be a reliable indicator of the prognosis of hepatocellular carcinoma [9]. However, the precise contribution of BCAA and lactate to multiple myeloma has not been elucidated yet.

In this study, we conducted an analysis of the characteristics of genes related to lactate and branched-chain amino acids in MM and constructed a risk signature associated with overall survival. First, MM samples were grouped into two distinct molecular subtypes according to BCAA and LMRG metabolism-related gene expression. Next, our analysis of RNA-seq data revealed 559 DEGs related to BCAA and LMRG between two molecular subtypes. In addition, we identified two genes (CKS2 and LYZ) as effective prognostic indicators through univariate Cox and LASSO regression. Next, patients can be divided into high-risk and low-risk groups according to risk scores. The result of K-M revealed that the prognosis is better for lower risks. The ROC curve demonstrates the effectiveness of the risk signature in predicting the survival rates of MM patients. Moreover, the independent prognostic value of the risk signature was verified through univariate and multivariate Cox analyses. Many metabolisms of branched-chain amino acids-related genes have proven to be effective prognostic biomarkers [30, 31]. Zheng et al. found that the metabolism of BCAAs is a key factor in the conversion of hematopoietic stem cells into leukemia [32]. Glushka et al. have also demonstrated that a modification of BCAA metabolism caused by the MSI2-BCAT1 axis is a contributor to the progression of myeloid leukemia [14]. Our research went beyond creating a risk signature that could effectively predict the OS of MM patients in both the training set and validation set. It was pointed out that these genes related to BCAA and LMRGs metabolism played a major role in the progression of MM.

Tumor Microenvironment has highlighted the importance of immune cells in the development of MM [33]. Cytotoxic T lymphocytes (CTLs) are the primary players in adaptive cellular immune responses, responsible for effector functions. The monoclonal immunoglobulin (idiotype; Id) secreted by myeloma cells is classified as a tumor-specific antigen. Some studies have indicated that Id-pulsed dendritic cells can generate Id-specific CD8^+^ CTLs, which can eradicate primary myeloma cells from patients [34–36]. However, our results found that the high-risk group had a limited infiltration of CTL, and DC cells, implying that the absence of immune cells can lead to the ineffectiveness of an anti-tumor effect. Previous research has demonstrated that mice that are not provided with BCAAs are unable to generate proper antibody and cytotoxic T cell responses [37]. Meanwhile, BCAAs supplementation can bolster the body’s defense system by increasing the activity of CD8^+^T cells and the production of DC IL-12 [38, 39]. We considered that regulating metabolic pathways and augmenting the adaptive immune system may be a viable approach to treating MM. Macrophages, a part of innate immunity, typically demonstrate M2-like characteristics in multiple myeloma, which include limited cytotoxicity, decreased antigen presentation, increased angiogenesis and T cell inhibition, thus suppressing the immune system [40]. Recently, Zhao et al. found that lactate directly activates the expression of macrophages M2 related genes through histone lactate modification [41]. Consistently, our results showed that macrophages M2 cells were significantly up regulated in high-risk with MM patients. We speculate that lactate may contribute to the progression of MM by influencing the immune system’s ability to evade detection. In conclusion, an aberrant expression of genes associated with BCAA and LMRG-related metabolism may enable immune evasion [42],thus targeting two metabolic pathways to modulate the immune microenvironment is a potential strategy for treating multiple myeloma.

Our research also revealed that multiple immunomodulators had significantly decreased expression in the high-risk group. Granzyme A (GZMA), a protein produced by cytotoxic lymphocytes, can activate Gasdermin B protein in a highly precise and efficient manner, thus augmenting the body’s antitumor immune response [43].CTLs are responsible for the production of PRF1, which is able to form holes in the membrane of the target cell and set off a series of processes that result in its destruction [44].ARG1 can effectively reduce arginine in serum by transforming it into citrulline and ornithine, which leads to a decrease in tumor cell growth in melanoma patients who did not respond to anti-PD-1 and CTLA-4 therapies, thereby exhibiting antitumor activity [45]. In addition, TNFRSF9, also known as 4-1BB and CD137, belongs to the tumor necrosis factor receptor superfamily and has been shown to promote the proliferation of CD8^+^T, CD4^+^T and NK cells, and to infiltrate these cells into tumors [46, 47]. Based on the above, high-risk patients may reduce the anti-tumor effect due to a decreased expression of immunomodulators. Thus, exploiting immunomodulators as a therapeutic target could be a viable option for managing high risk MM.

Stratification survival analysis revealed that the risk signature possessed an accurate predictive value for prognosis in MM subtypes sorted by risk score, gender, IDH, chromosome abnormal, and ISS1 stage. Meanwhile, the clinic correlation analysis confirmed that the risk score was highly associated with clinical staging and high-risk markers. Chromosome 1q amplification is a common genetic alteration that is seen in multiple myeloma, and is believed to be indicative of a high-risk [48]. The prognosis model created by risk score indicates that the proportion of a 1q chromosome deletion is higher in the high-risk group. Evidence suggests that the deletion of chromosome 13q14 is associated with the emergence and progression of multiple myeloma [49]. Consistently, our study verified that patients with a 13q chromosome deletion are more likely to be classified as high-risk. The International Staging System (ISS) is a commonly employed MM staging system in clinical practice, providing a more precise evaluation of the prognosis of patients. Our analysis has demonstrated that the -high-risk group has a higher proportion of ISS III patients and a lower proportion of ISS I patients than the low-risk group. In addition, the ROC curves for the 2-BCAA and LMRG related gene signature in both the training and testing cohorts had AUC values that were significantly higher than 0.65 for 1 and 5-year periods. These results demonstrated that the risk model created by BCAA and LMRG is a reliable indicator of the prognosis of MM.

We identified that LMRG and BCAA-related prognostic DEGs, in MM patients, statistically correlated with the overall survival by K-M survival analysis. GSEA analysis revealed that the DEGs in the high-risk group were mainly associated with genes encoding cell cycle-related targets of E2F transcription factors, a subgroup of genes that are regulated by MYC. MYC mutations are hypothesized to be the source of undetermined significance to MM transition, as well as a late genomic event that is responsible for tumor progression [50]. To date, studies have revealed that MYC can augment the expression of genes that facilitate the uptake of nutrients such as glucose and glutamine, in order to generate ATP and absorb the fundamental components of the cell, thereby inducing the replication of DNA and cell division [51].TP53 deletion in Burkitt lymphoma leads to an overexpression of MYC, subsequently leading to an overabundance of nutrients consumption [52].Research has demonstrated that bromodomain proteins inhibition, inhibition of MYC translation and ribosomal biogenesis and targeting the immune microenvironment are beneficial in treating myeloma due to their effect on MYC [53, 54]. Several drugs targeting MYC have been identified for Multiple Myeloma and have been evaluated in clinical trials [55, 56]. Importantly, the high-risk group exhibited a significantly higher CKS2 level than the low-risk group. CKS2, a cyclin-dependent kinase subunit, has been identified as playing an important role in both cell cycle and cell proliferation [57]. JH Xu et al. indicated that CKS2 might function as a tumor promoter and could serve as a promising prognostic biomarker for epithelial ovarian cancer [58]. Although, previous research has demonstrated that there is no association between CKS2 expression and osteolytic bone disease in MM. However, we found that knockdown of the CKS2 inhibited MM cell viability, migration, and invasion potential and promoted cell apoptosis. It can be regarded as a potential target for anti-MM therapy or as a molecular marker of the metabolic abnormal state in our research.

Numerous patients who have been exposed to proteasome inhibitors, immunomodulatory drugs, and monoclonal antibodies targeting CD38 have become unresponsive to at least one of these treatments [59]. Thus, novel drugs with dissimilar methods of action are required. Through AutoDock’s molecular docking process, it was determined that CKS2 and LYZ possess the highest sensitivity to Temozolomide_1375. MM is characterized by frequent chromosomal instability and dysfunctional DNA repair [60]. To enhance the potency of genotoxic therapy, suppressing DNA repair is an effective choice [61]. Temozolomide(TMZ) induces single-stranded breaks, halts cell division, and triggers apoptosis [62]. It is utilized to treat glioma and leukemia [63, 64]. Hong-Yuan Shen et al. demonstrated that TMZ promoted DNA damage, cell cycle arrest, and apoptotic death in human MM cells and xenograft mice model [65]. Recent research has shown that melflufen, a novel alkylator, is highly effective in treating MM [66]. They have demonstrated that melflufen and other treatments such as selinexor, venetoclax, belantamab, mafodotin, and, adoptive immunotherapy can significantly improve the life expectancy of individuals suffering from MM [67]. However, the optimal order and the most effective way of treatment remain undetermined. Therefore, prior to administering treatment to individuals with MM, it is essential to consider the patient’s profile, which includes their metabolic and other related indicators. Based on the our results, we suggested that Temozolomide may be a viable option for addressing the abnormal levels of branched chain amino acid and lactate in myeloma patients.

## Conclusions

In the study, our research has identified genes related to branched-chain amino acids and lactate metabolism as potential prognostic biomarkers and has developed a novel risk signature that is independently associated with the overall survival of multiple myeloma patients. We conducted an analysis of two genes that are related to branched-chain amino acids and lactate metabolism as a predictive marker and established its effectiveness in risk stratification. Nevertheless, our research is unavoidably subject to certain limitations. First, the risk signature was identified and validated through analysis of GEO and TCGA datasets. The efficacy of the BCAA and LMRG metabolism-related signature as a prognostic indicator for MM patients has yet to be established due to the lack of our own relevant data. In order to ensure accuracy, external validation should be conducted using our own clinic data in the future. Moreover, to estimate the proportion of immune cells, bioinformatics analysis was utilized instead of direct measurements from peripheral blood and animal models, which may not be entirely accurate. Future research will involve clinical and laboratory experiments to confirm the precise role of the risk signature in the success of immunotherapy for MM patients.

### Electronic supplementary material

Below is the link to the electronic supplementary material.

**Additional file 1: Table S1** Primer sequences

**Additional file 2: Table S2** Differential IC50s observed for 168 drugs in two groups

**Additional file 3: Table S3** siRNA sequences

**Additional file 4: Figure S1** Assessment of the prognostic risk signature in TCGA. **A** The distributions of risk score, survival status and expression profile of signature genes between the risk groups. **B** K-M survival analysis between the high- and low-risk groups. **(C)** ROC curve at 1-, 3- and 5-years of prognostic value of the prognostic index

**Additional file 5: Figure S2** Assessment of the prognostic risk signature in GSE136337. **A** The distributions of risk score, survival status and expression profile of signature genes between the risk groups. **B** K-M survival analysis between the high- and low-risk groups. **C** ROC curve at 1-, 3- and 5-years of prognostic value of the prognostic index

**Additional file 6: Figure S3** Assessment of the prognostic risk signature. **A** K-M survival analysis in Subgrp7-CD1 between the high- and low-risk groups in training set and TCGA. **B-F** K-M survival analysis in OS, iss1, gender, del17pcyto, and del16qclinicalfish between the two risk groups in GSE136337

**Additional file 7: Figure S4** Effects of Nelarabine, Carmustine and Temozolomide on apoptotic death, invasion and migration in MM cells. **A** MM1.S cells were administered Nelarabine (5 μm), Carmustine (300 μm) and Temozolomide (50 μm) for 24 h, and apoptosis was examined stained with annexin V/FITC and 7AAD (4 A Biotech). **B** Invasion and migration ratio of MM cell toward four groups through Transwell membranes (5-mm pore size) were assessed. Independent experiments were performed 3 times. n = 5 per group (**, p < 0.01; ****, p < 0.0001; vs. CONTROL; ns, no significance)

## Data Availability

The public data used in this study can be found from the Gene-Expression Omnibus (GEO; https://www.ncbi.nlm.nih.gov/geo/) database. All other data supporting the findings of this study are available within the paper. The raw data that support the findings of this study are available from the corresponding author upon reasonable request.

## Abbreviations

MM: multiple myeloma
LMRGs: lactate metabolism-related genes
LDH: lactate dehydrogenase
BCAAs: branched chain amino acids
PCA: principal component analysis
OS: overall survival
GO: Gene Ontology
KEGG: Kyoto Encyclopedia of Genes and Genomes
TISCH: Tumor Immune Single-cell Hub
UMAP: Uniform Manifold Approximation and Projection
GEO: Gene Expression Omnibus
MSigDB: Molecular Signatures Database
MCPcounter: Microenvironment Cell Populations-counter
OD: Optical Density
CTL: cytotoxic T lymphocyte
GZMA: granzyme A
ISS: International Staging System
TMZ: Temozolomide
