# Supplementary figures and images for: Characterization and application of a lactate and branched chain amino acid metabolism related gene signature in a prognosis risk model for multiple myeloma
Source: Cancer Cell Int. 2023 Aug 14;23:169. doi: 10.1186/s12935-023-03007-4 (PMC10426219; doi:10.1186/s12935-023-03007-4)

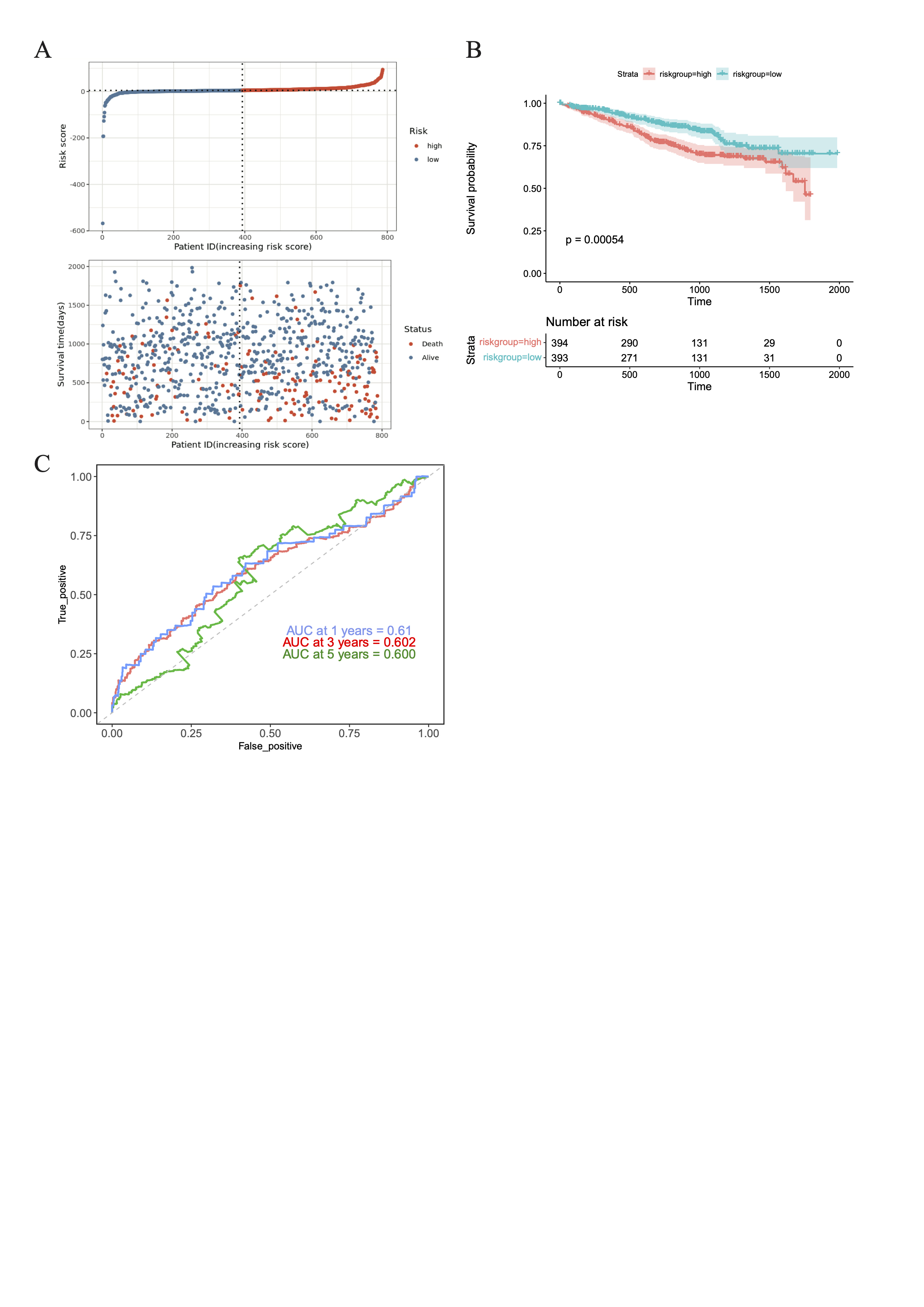

Supplement: Supplementary file 4 — Additional file 4: Figure S1 Assessment of the prognostic risk signature in TCGA. A The distributions of risk score, survival status and expression profile of signature genes between the risk groups. B K-M survival analysis between the high- and low-risk groups. (C) ROC curve at 1-, 3- and 5-years of prognostic value of the prognostic index [file 12935_2023_3007_MOESM4_ESM.jpg]

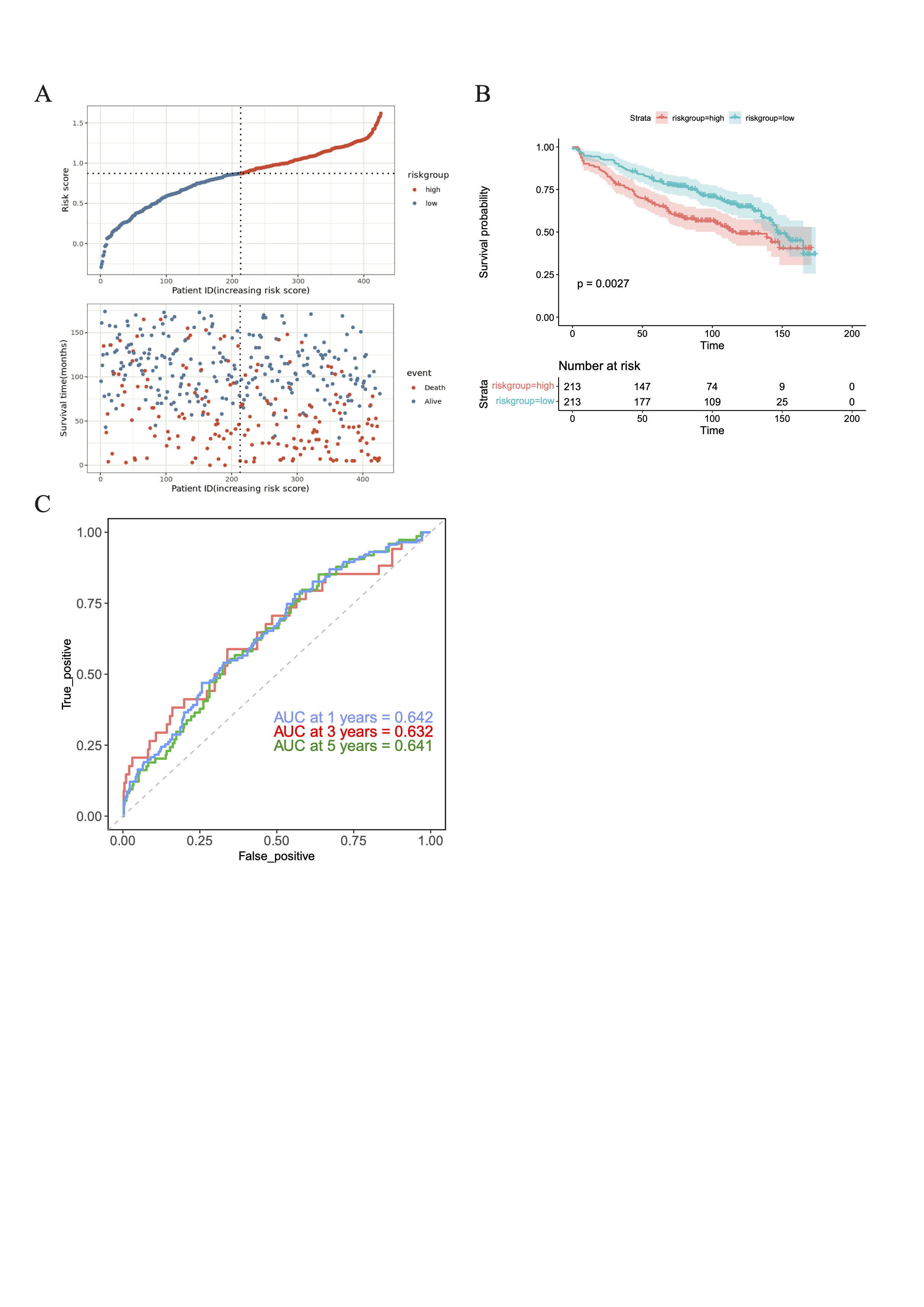

Supplement: Supplementary file 5 — Additional file 5: Figure S2 Assessment of the prognostic risk signature in GSE136337. A The distributions of risk score, survival status and expression profile of signature genes between the risk groups. B K-M survival analysis between the high- and low-risk groups. C ROC curve at 1-, 3- and 5-years of prognostic value of the prognostic index [file 12935_2023_3007_MOESM5_ESM.jpg]

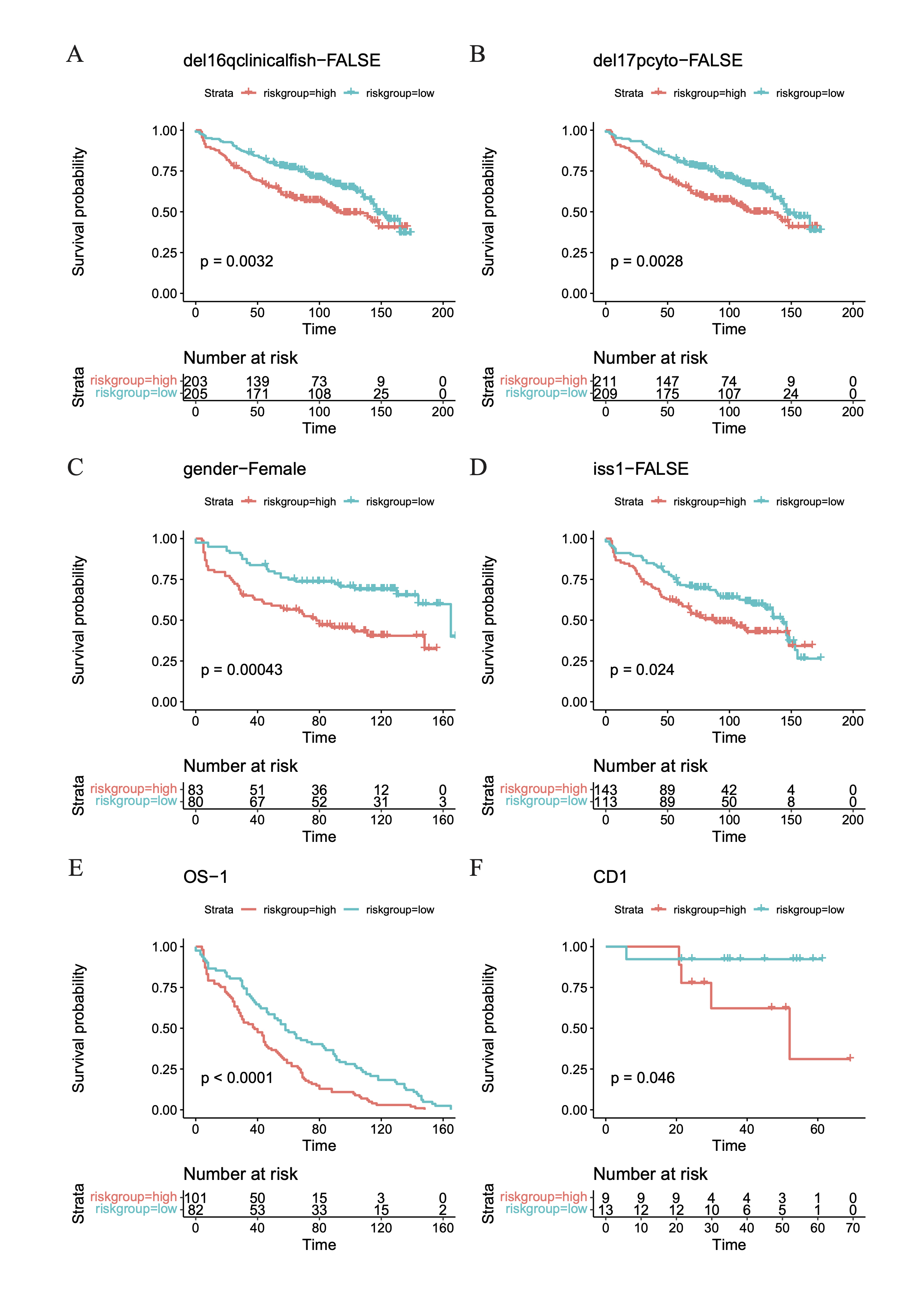

Supplement: Supplementary file 6 — Additional file 6: Figure S3 Assessment of the prognostic risk signature. A K-M survival analysis in Subgrp7-CD1 between the high- and low-risk groups in training set and TCGA. B-F K-M survival analysis in OS, iss1, gender, del17pcyto, and del16qclinicalfish between the two risk groups in GSE136337 [file 12935_2023_3007_MOESM6_ESM.jpg]

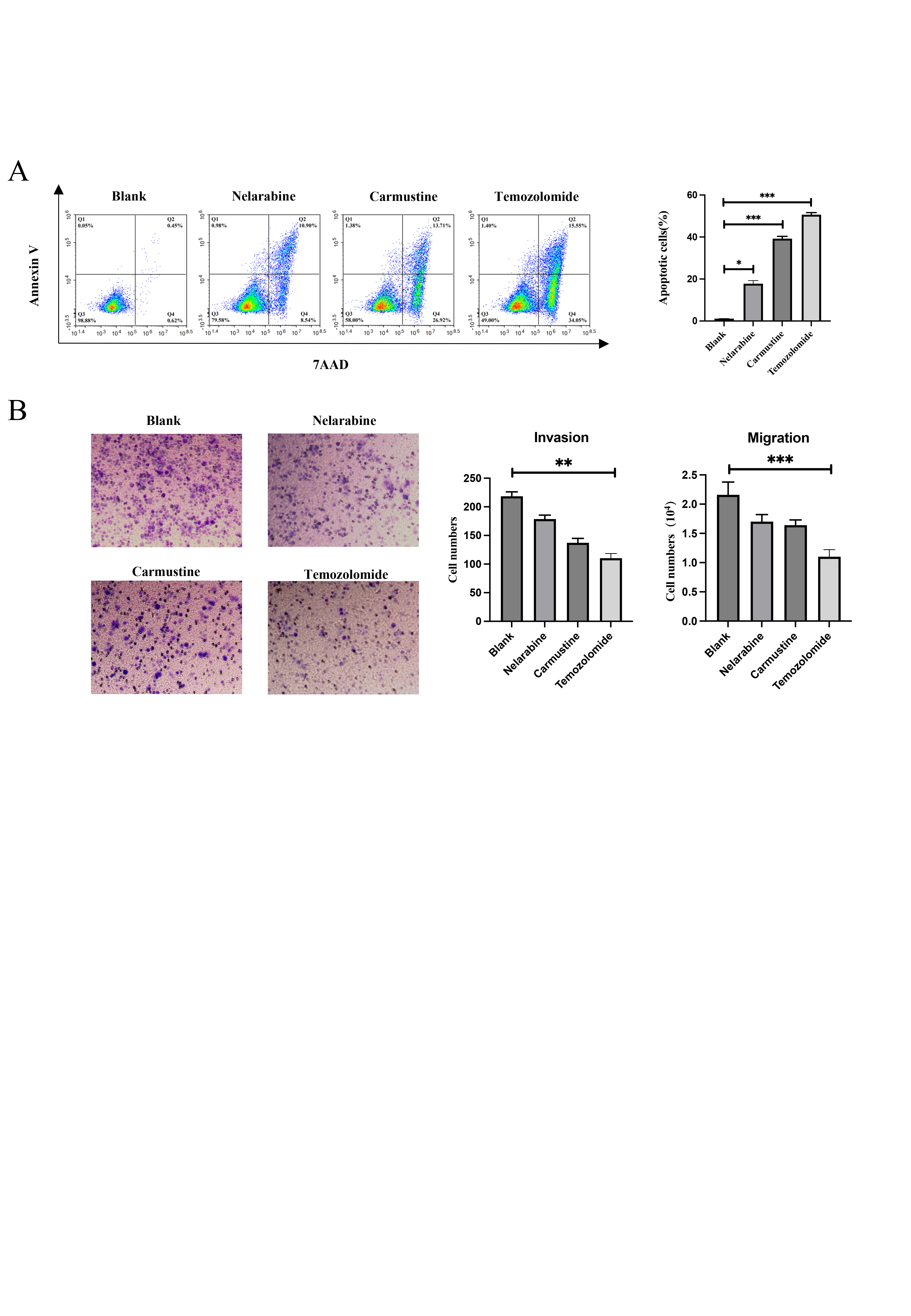

Supplement: Supplementary file 7 — Additional file 7: Figure S4 Effects of Nelarabine, Carmustine and Temozolomide on apoptotic death, invasion and migration in MM cells. A MM1.S cells were administered Nelarabine (5 μm), Carmustine (300 μm) and Temozolomide (50 μm) for 24 h, and apoptosis was examined stained with annexin V/FITC and 7AAD (4 A Biotech). B Invasion and migration ratio of MM cell toward four groups through Transwell membranes (5-mm pore size) were assessed. Independent experiments were performed 3 times. n = 5 per group (**, p < 0.01; ****, p < 0.0001; vs. CONTROL; ns, no significance) [file 12935_2023_3007_MOESM7_ESM.jpg]
